# A Qualitative Investigation of Preparedness for the Launch of 988: Implications for the Continuum of Emergency Mental Health Care

**DOI:** 10.1007/s10488-023-01263-0

**Published:** 2023-03-29

**Authors:** Stephanie Brooks Holliday, Samantha Matthews, Armenda Bialas, Ryan K. McBain, Jonathan H. Cantor, Nicole Eberhart, Joshua Breslau

**Affiliations:** RAND Corporation, 1776 Main Street, 90405 Santa Monica, CA USA

**Keywords:** Crisis, Suicide prevention, Mental health, Services

## Abstract

On July 16, 2022, the 988 mental health crisis hotline launched nationwide. In addition to preparing for an increase in call volume, many jurisdictions used the launch of 988 as an opportunity to examine their full continuum of emergency mental health care. Our goal was to understand the characteristics of jurisdictions’ existing continuums of care, identify factors that distinguished jurisdictions that were more- versus less-prepared for 988, and explore perceived strengths and limitations of the planning process. We conducted 15 qualitative interviews with state and local mental health program directors representing 10 states based on their preparedness for the 988 rollout. Interviews focused on 988 call centers, mobile crisis response, and crisis stabilization, as well as strengths and limitations of the 988 planning process. Data were analyzed using rapid qualitative analysis, an approach designed to draw insights on evolving processes and extract actionable findings. Interviewees from jurisdictions that reported that they were more-prepared for the launch of 988 tended to have local 988 call centers and already had local access to mobile crisis teams and crisis stabilization units. Interviewees across jurisdictions described challenges to offering a robust continuum of crisis services, including workforce shortages and geographic constraints. Though jurisdictions acknowledged the importance of integrating peer support staff and serving diverse populations, many perceived room for growth in these areas. Though 988 has launched, efforts to bolster the existing continuum will continue and hinge on efforts to expand the behavioral health workforce, engage diverse partners, and collect relevant data.

## Introduction

The 988 Suicide and Crisis Lifeline was established by the National Suicide Hotline Designation Act of 2020 (which became Public Law Number 116–172 in October 2020) and launched about 24 months later, on July 16, 2022 (Gardner, 2020). This three-digit number was designed to replace the existing National Suicide Prevention Lifeline number (1-800-273-TALK) with an easier-to-remember alternative. Research on the Lifeline and other emergency crisis lines has found that these hotlines can reduce distress and suicidality, keep people safe, and provide referrals that many callers follow-up on after the call (Matthews et al., 2022). In this way, the transition to 988 was designed with the goal of expanding access to this importance service and emergency mental health care, as well as reducing stigma related to seeking such help.

Callers who dial 988 reach the existing Lifeline network, which comprises more than 200 crisis centers across the country (988 Suicide & Crisis Lifeline, n.d.). In preparing for the launch of 988, Vibrant Emotional Health (Vibrant) – the company that operates the Lifeline network – projected that the volume of calls could increase compared to pre-implementation levels by as much as 50% within the first year (Vibrant Emotional Health, 2020). A statement from Vibrant reported a 45% increase in the volume of calls, texts, and chats to the Lifeline during the week that 988 launched compared to the prior week (Quintana, 2022).

In addition to preparing for an increase in call volume, many jurisdictions used the launch of 988 as an opportunity to examine their full continuum of emergency mental health care (SAMHSA, 2021b). Prior to the launch of 988, anecdotal estimates suggested that as many as 80% of calls to emergency mental health hotlines could be resolved during the telephone call – for example, by providing an intervention via telephone or connecting the caller with local resources. However, a certain subset of callers require an in-person response, necessitating that jurisdictions have relevant emergency mental health care services available. Therefore, as part of the process for preparing for 988, jurisdictions have been encouraged not only to ensure that they have a well-staffed call center, but also to examine their continuum of crisis services and identify opportunities for improvement.

The ideal mental health crisis response system has been described as having three key components: crisis call centers, or “someone to talk to;” mobile crisis teams, or “someone to respond,” and crisis stabilization programs, or “somewhere to go” (NAMI, n.d.; SAMHSA, 2020). There are several sources of guidance on the key features of this emergency mental health care continuum, including guides by the Substance Abuse and Mental Health Services Administration (SAMHSA) and National Alliance on Mental Illness (NAMI). For example, this guidance indicates that crisis call centers should operate 24 h a day, seven days a week (24/7), and be staffed by people who are trained in suicide risk assessment and intervention and are aware of resources local to the caller. Mobile crisis teams are ideally available 24/7 and staffed with clinicians. Though law enforcement may be a partner to such mobile crisis teams, mental health professionals and peer support staff should be the lead responders. Finally, crisis stabilization programs should accept multiple types of referrals (e.g., walk-ins, drop-off by law enforcement or mobile response teams) and be staffed 24/7 with mental health professionals, medical staff, and peer support staff.

Many national organizations have provided guidance to jurisdictions seeking to align their crisis continuum with best practices (e.g., National Association of State Mental Health Program Directors [NASMHPD) [n.d.]), Pew Charitable Trusts; Wertheimer & Angelone (2022), National Association of Counties; Thomson (2022); Well Being Trust (2021). At the same time, jurisdictions have faced important obstacles to bolstering their mental health emergency continuum of care. Though the National Suicide Hotline Designation Act enables states to pass their own legislation to establish a surcharge to fund 988 operations, only four states had done so as of July 16, 2022 (National Academy for State Health Policy, 2022). Though other funds have been made available through planning and capacity building grants and American Rescue Plan allocations (HHS, 2021), many jurisdictions have expressed concern about whether they have enough funds to bolster their mental health infrastructure (Ohio Department of Mental Health and Addiction Services, 2021; Rhode Island Department of Behavioral Healthcare, Developmental Disabilities & Hospitals, n.d.). In addition, the organization of mental health services varies across states and localities, making it challenging to apply uniform standards, such as those developed by SAMHSA and NASMHPD.

## Current Study

To better understand how jurisdictions were preparing for the launch of 988, we conducted a series of qualitative interviews with state and local mental health program directors in the months leading up to July 16, 2022. Our goal was to understand the characteristics of jurisdictions’ existing continuums of care and any plans or changes they were making to their continuum of care in anticipation of 988 implementation, and to explore perceived strengths and limitations of their 988-planning process. We were particularly interested in understanding the experience of jurisdictions who indicated that they were well-prepared based on their responses to a survey fielded in an earlier phase of this study, as well as those who indicated that they were least well-prepared. This enabled us to also explore features of the continuum of care and 988 preparation process that were common in both more and less-prepared jurisdictions, as well as factors that differentiated more- and less-prepared jurisdictions. This study fills an important gap in the literature, as it not only reveals key information about the process of preparing for 988 implementation, but highlights the ongoing challenges and efforts of jurisdictions as they work to bolster their continuum of emergency mental health care.

## Methods

### Participants and Procedures

Data were collected as part of broader study examining state and county preparedness for the implementation of 988. During the first phase of the study, we surveyed programmatic leads of public-sector mental health agencies at state, regional, and county levels. Of the 688 state and county mental health program directors invited to participate in the survey, 180 completed the survey (for more detail on the survey, please see Cantor, et al., 2022).

To complement the survey, we were interested in gaining an in-depth understanding of the ways that counties and states have been planning for 988 implementation. We therefore conducted semi-structured interviews with a subset of respondents who completed the survey and who agreed to be contacted for an in-depth interview.

To identify potential interviewees, we included a final item on the survey asking respondents to indicate if they would be open to participating in a brief follow-up interview to discuss their experiences with 988. In total, 23 survey respondents from 10 states indicated they were willing to be contacted about an interview (about 13% of survey respondents). We characterized each jurisdiction’s 988 implementation preparedness using a subset of survey items, including items asking how prepared they were with respect to staffing, financing, infrastructure, and coordination of services. Jurisdictions indicating they were “somewhat” or “very” prepared on two or more of the four domains were classified in the more-prepared group, while those indicating they were “not at all prepared” on two or more domains were classified in the less-prepared group.

When selecting potential interviewees, we were also careful not to overrepresent a single state, especially as there were only 10 states represented among potential interviewees. Therefore, we interviewed a maximum of two individuals from a given state. We also examined the population of the jurisdiction each potential interviewee represented, with the goal of obtaining variation with respect to population size and geography. We selected these characteristics because population size, urbanicity, and other geographic features can influence the availability of mental health resources and affect residents’ ability to access those resources. In total, we invited 17 individuals to participate in interviews, nine from less-prepared jurisdictions and eight from more-prepared jurisdictions. Of those, 15 agreed to participate in interviews: eight from less-prepared and seven from more-prepared jurisdictions. This included one interviewee from a state-level agency and 14 from county-level or regional agencies (i.e., agencies serving more than one county). We had representation from each of the 10 states, including five states that contributed two interviewees and five states with one interviewee.

Interviews lasted up to 45 min, and interviewees were eligible to receive a $50 gift card for their participation (not all interviewees were able to accept a gift card due to constraints of their government employment). Each interview included two project team members: one lead interviewer and one notetaker. Interviews were recorded; we took detailed notes during the interviews and used the recordings to fill in detail and identify verbatim quotes for analysis. All procedures were approved by the RAND Corporation Institutional Review Board.

### Measures

We developed a semi-structured interview protocol focused on six topics. The first section requested background information on the interviewee, their agency, and the agency’s role in preparing for 988. The next three sections were structured around the continuum of emergency mental health services, asking about planning and preparedness related to (a) the 988 hotline (“someone to call”), (b) mobile emergency teams (“someone to respond”), and (c) emergency receiving and stabilization services (“somewhere to go”). The fifth section centered on questions related to resources, staffing, and equity considerations. The final section focused on overall strengths and limitations of the 988 planning process.

In addition to centering on the continuum of emergency mental health services, interview questions were designed to be complementary to the survey items. For example, the survey asked respondents to indicate whether they had a mental health emergency hotline and, if so, whether it was part of the Lifeline network. Interviewees from jurisdictions with a regional Lifeline network call center were then asked questions about whether the call center was prepared for the launch of 988, how they anticipate the launch to affect call volume, and challenges they have experienced in preparing their call center. Interviewees from jurisdictions without a regional Lifeline call center were asked how 988 calls from their jurisdiction will be handled and to describe any challenges they foresee.

### Analysis

Our goal with analysis was to quickly extract policy-relevant, timely findings from jurisdictions, understanding that many jurisdictions’ 988 planning was evolving in the lead-up to July 16th. We analyzed the interviews using rapid qualitative analysis (RQA), (Taylor et al., 2018) an approach that enabled us to extract key findings as interviews were conducted. RQA is well-suited to research on topics that are quickly evolving, where the goal is explain a process or phenomenon and extract actionable findings (Taylor et al., 2018). We developed a coding spreadsheet that reflected our key themes of interest, which were largely identified deductively based on the interview protocol (e.g., “guidance used to prepare for 988,” “agencies collaborating for the launch of 988”). After each interview, the lead interviewer extracted key findings related to each theme and summarized them in the analysis spreadsheet, and the notetaker reviewed the coding to ensure completeness and accuracy. The coding team met regularly during analysis to discuss emerging themes and consistent coding.

Given our purposeful selection criteria (i.e., representatives from more- and less-prepared jurisdictions) and self-selection into the pool of potential interviewees, analysis focused on describing the range of responses that were expressed (Levitt, 2018; Maxwell, 2010; Neale et al., 2014). This included efforts to identify common responses and to examine whether findings were different between the more-prepared and less-prepared jurisdictions. We also identified illustrative quotes.

## Results

### Process of Planning for 988

#### Role of Agency

We asked interviewees to share their role in preparing for 988. As leaders of mental or behavioral health agencies, interviewees discussed roles related to coordinating with other agencies at the state, regional, and local levels. At the state level, four interviewees participated in a statewide 988 planning committee or task force, though doing so was not always associated with a greater feeling of preparedness for the transition to 988. At the regional level, county-level officials often coordinated with other county directors. At the local level, interviewees often contracted with crisis service providers, and with these providers, oversaw the development or expansion of services in preparation for 988.

#### Collaborations in Support of 988 Implementation

Jurisdictions collaborated with a range of diverse agencies in planning for the transition to 988. Nearly all had been in communication with state and county government agencies, particularly Departments of Mental or Behavioral Health, Human Services, and law enforcement agencies. Outside of government agencies, interviewees frequently emphasized the importance of collaborating with local service providers, often relying on their expertise in the strategic planning process. These organizations included crisis line operators and crisis service providers, Certified Community Behavioral Health Clinics (CCBHCs), hospitals, and homeless service providers and shelters. In planning for 988, interviewees from better-prepared jurisdictions tended to describe collaborations with a local crisis line operator or mobile crisis team provider. One interviewee emphasized that they expected strong collaborations between agencies to “make all the difference” in implementing 988. Another interviewee offered the following advice to other jurisdictions for 988 planning:

It’s really just making sure all those key stakeholders are talking and planning from day one. Sometimes government and different parts of governments operate in siloes, so by us talking earlier on we were able to connect some of the pieces, look at some of the gaps in funding, just really coordinating this together with police and all the key stakeholders. It is really that partnership that is so crucial. (14, County)

#### Sources of Guidance Used for 988 Planning

Jurisdictions relied on a range of sources of guidance for planning the transition to 988. Most commonly, interviewees received guidance from a state-level department of mental or behavioral health or from county directors of mental or behavioral health. Interviewees also highlighted a variety of national sources of information including SAMHSA, Vibrant, and the National Council for Mental Wellbeing—specifically, their Roadmap to an Ideal Crisis System.

Although interviewees relied on different sources for guidance, a few interviewees expressed concern with the lack of consistent guidance provided by their state (as noted by county and regional officials) or the federal government. This concern was shared by interviewees from both more-prepared and less-prepared jurisdictions. For example, one interviewee, who had indicated their jurisdiction was more prepared for 988, stated:

I don’t feel like there was a lot of federal guidance on this, and I feel like there should have been more. We have a federal initiative being handed down, and we were told that – I felt like we were handed coloring sheets and a box of crayons, and [it was like they said] ‘here you go.’ I think when you’re rolling out something like this, when it affects our folks with mental health, we have to be, we have to do it with intention, and we have to make sure that people are consistent. (9, Region)

Another interviewee, from a less-prepared jurisdiction, noted that they understand the vision for 988 and the ideal continuum of emergency mental health care, but have a hard time knowing how to reconcile that with their local resources, policies, and practices:

I feel like I have a reasonable grasp of what [988] is meant to be, and then I have a reasonable grasp of our local function of what we do and how we do it, and then I just have a giant disconnect between the concept over here [of 988 and the continuum of services] and the reality over here. (8, County)

Other county and regional representatives expressed a need for more lines of communication and data and information sharing from their state, as well best practices from other states and counties, including rural and suburban areas.

### Someone to Call: Preparedness Related to 988 and the Lifeline Network Call Centers

#### Call Center Location and Structure

Key themes related to the continuum of care are summarized in Table 1. Agencies varied with respect to how calls to 988 would be handled and the structure of their call centers. Some interviewees noted that their county or region had a local Lifeline network call center, whereas others relied on a call center in a different county or region or a call center operated at the state level. Administrators in jurisdictions that already possessed a local Lifeline operator, or had a local hotline recently become a Lifeline operator, noted this as a benefit for 988 preparedness. For example, they felt more confident that Lifeline staff would be aware of local resources, knew that staff were already prepared to handle the types of calls that will be made to 988, and were able to engage in planning for the interface between 911 and 988.

**Table 1:** Summary of key themes related to the continuum of care

Point on the continuum of care	Common themes
Someone to call	• Most better-prepared jurisdictions had a local call center and half had a local Lifeline center. These jurisdictions saw this as a benefit for 988 preparedness and felt more confident that call center staff are aware of local resources. • A key concern of all jurisdictions was related to call center capacity and workforce. • Challenges to providing call center services included difficulties predicting changes in call volume and types of calls, coordinating 988 and 911, ensuring adequate training for 911 staff, and educating the public on the purpose of 988.
Someone to respond	• Many well-prepared and half the less-prepared jurisdictions had 24/7 mobile crisis teams that can respond within 30 min • When jurisdictions did not have mobile crisis response, law enforcement was often the default responder • Some jurisdictions had clinician-led teams, but some collaborated with law enforcement (e.g., through co-responder models) • Challenges to providing mobile crisis services included workforce shortages, burnout, and geographic constraints
Somewhere to go	• About half of jurisdictions had crisis stabilization services, most of which were better-prepared counties • Establishing crisis stabilization units often took many years • Barriers to providing crisis stabilization services included geographic constraints and funding constraints, and a lack of volume needed to justify 24/7 units • When jurisdictions did not have a crisis stabilization unit, emergency departments were often the default care option • It was seen as important to think beyond crisis stabilization to pre- and post-crisis care (e.g., outpatient services)

By contrast, jurisdictions that relied on a regional or statewide call center expressed concern that Lifeline staff would not be aware of local resources, with one interviewee describing the state-level or regional call centers as “disconnected” from county operations. It was also more challenging for those interviewees to comment on how prepared those regional or statewide call centers were for the transition to 988, as they had less visibility into the planning and preparedness process. However, some did express concern about the preparedness of these state or regional call centers; for example, one interviewee described the transition to 988 as “an extremely large lift” (4, Region) for their centralized call center, and another noted that they were not “100% confident” their call center would be prepared because “change is very hard for people” (5, Region). Another interviewee noted that, if calls will be mostly resolved over the phone, they expected that their regional center will be prepared; however, if their local call center needed to connect to local systems and services (e.g., to make referrals and handoffs to care), “it’s multiple counties, geographies, and clinical realities away.” (8, County).

More-prepared agencies described the ways that their local hotlines were preparing for 988 implementation. For example, some longer-standing call centers had recently received funding to prepare for 988, including grants from Vibrant. One agency had recently applied for funding to develop a call center, and another had been leading efforts in their state legislature to advance 988 legislation and expressed the importance of having a state legislature that is committed to funding crisis lines with bipartisan support.

#### Call Center-Related Challenges and Concerns

A key concern of both more- and less-prepared jurisdictions and all types of call centers – local, regional, and statewide – related to **capacity and workforce**. Although interviewees noted difficulty with projecting changes in volume and therefore predicting staffing needs, many were expecting increases in calls and looking to expand capacity and hire additional staff. Many jurisdictions had hired or had plans to hire additional staff with funding from their state or through grants, including CCBHC and Vibrant grants. However, many interviewees shared struggles with recruitment, training, and retention, exacerbated by the COVID-19 pandemic.

With additional funding, some jurisdictions had also worked to develop **projections for future call trends**. Some more-prepared jurisdictions noted the importance of looking at data on call volume, caller characteristics, and reasons for calling in assessing changes in call volume and staffing needs. One interviewee shared their approach to developing projections for both call and in-person response volume, specifically working with Vibrant and using the Crisis Now Crisis Resource Need Calculator. That interviewee shared:

We worked with Vibrant to come up with state projections for Fiscal Year 23… That’s kind of what we’ve used to help make estimates on our amount of funding needed and our staffing patterns using the Erlang C methodology for that. I think you know we also sort of identified that above and beyond just the ability to answer the phones, we’ve got a lot of capacity building that needs to occur in [the jurisdiction] around the crisis services as well. We’ve been using the Crisis Now model calculator to help figure out what our target numbers for that might be in terms of how many crisis teams do we need and how much funding we need for that. (2, State)

However, some jurisdictions reported challenges predicting whether the proportion of calls to 988 that could be resolved over the phone versus requiring an in-person response would change.

Many interviewees raised concerns around **existing call centers and crisis lines** and their transition to or connection to 988. Some jurisdictions had plans to eventually transition to only using 988 for mental health emergency calls, and others had plans to keep their existing local phone number operational but route calls to Lifeline network call centers to avoid confusion among residents. Some also raised questions about how their non-Lifeline local call center would interface with 988 when it goes live.

Interviewees from certain jurisdictions raised the topic of **planning for service coordination between 988 and 911**. Some interviewees noted a lack of concrete plans but said that they appreciate the importance of education and training for 911 staff. One interviewee noted the importance of having 911 staff “rethink and retrain” their response to behavioral health emergencies and said:

We have been working with our emergency communications center. We’ve done some trainings for them to help better identify a mental health call and to consider at times not sending the police initially but to connect it directly to [our crisis services]. That’s a slow process because it’s just 911, they deal with it the way they deal with it. We’re slowly trying to get them to understand that, and I think 988 will help because in their mind 988 is akin to 911 so they might be much more willing to switch to that than directly to [our crisis services]. (11, County)

By contrast, an interviewee from a less-prepared jurisdiction said there had been local resistance to connecting 911 and 988 along with difficulties of getting local buy-in for 988 and clinician-only response, or response without law enforcement, and saw this as a limitation to their crisis response model.

Finally, interviewees expressed uncertainty about **public education and marketing of 988**. Some interviewees described local plans to market use of 988, such as through county websites and social media, or partnering with their region’s faith-based community as “credible messengers” of 988 information. However, others felt there was little guidance on public education—specifically, whose role it is to educate the public and how to best do so. There was uncertainty regarding what would be publicized at the state and national levels, and in turn how that might affect call volume. An interviewee, from a more-prepared jurisdiction, shared:

There’s a lot of concern about how we market [988] and how it’s going to be marketed and what the purpose is and is that going to be clear to average citizen or consumer out there. (6, County)

### Someone to Respond: Preparedness Related to Mobile Crisis Response

#### Mobile Crisis Response Structure

Interviewees varied with respect to their handling of mobile crisis response. Most of the more-prepared counties and half of the less-prepared counties reported that they do have mobile crisis response teams that are available 24 h a day, seven days a week (24/7). Most of these jurisdictions had teams working 24/7, though some used a 24/7 on-call model. In some more-prepared counties, interviewees highlighted that their teams are typically able to respond within 30 min and serve all areas of their county. There were also a small number of jurisdictions that reported that they have mobile crisis response teams, but have not yet reached 24/7 coverage; interviewees from these jurisdictions also noted that they haven’t been able to reach all areas of their county, as they have rural areas that are more difficult to reach.

Interviewees from three less-prepared jurisdictions and one more-prepared jurisdiction reported that they have no mobile crisis response. Interviewees from most of these jurisdictions noted that often law enforcement responds to individuals experiencing mental health crises and transports them to the local hospital. There were efforts to try to extend the range of crisis services in other ways; for example, one jurisdiction had crisis staff who could respond to the local hospital or jail if someone was experiencing a mental health emergency. Another county had a staff member of their local behavioral health provider who had informally filled a “mobile crisis” role for the community, sometimes responding to people’s homes or following up after a hospitalization. They acknowledged that this arrangement is not sustainable, though.

#### Staffing

Jurisdictions varied with respect to the staffing of their mobile crisis teams. Some interviewees noted that their jurisdiction has a primarily clinician-led team. Though the “gold standard” for mobile crisis teams is to include a master’s level clinician, some of these clinician-led teams included a bachelor’s level clinician as the lead clinician. This reflected larger workforce-related challenges with hiring and retaining clinical staff. As one interviewee stated:

As you know there’s staffing issues throughout the state, so our mobile crisis has needed to get a little creative in terms of their hiring for this service. So sometimes the person accompanying isn’t a master’s level clinician. So there’s a lot of additional training that goes into that.” (5, Region)

Interviewees from most jurisdictions reported that, to date, law enforcement has had a role in mobile crisis response and they have been collaborating with law enforcement as part of their 988 implementation planning process. Some interviewees noted that law enforcement officers in their jurisdiction have received Crisis Intervention Team (CIT) training (Franz & Borum, 2011), which prepares them to respond to situations involving individuals with mental illness. Some have formal co-responder models, which pair law enforcement officers with clinicians, and several interviewees noted that their behavioral health agency has a good working relationship with local law enforcement. However, many interviewees raised questions about the ideal role for law enforcement in mobile crisis response. For example, one interviewee noted that co-responder models tend to be popular in their states, but they said they ultimately hope that they develop a model “where law enforcement is a backup, and not the primary response” (5, Region). Another said that they conceptualize it as a “Venn diagram” of police and behavioral health staff, nothing that “law enforcement has a need that’s broader than actually behavioral health crisis…and then we have lots of situations where we have no need for law enforcement, ideally. And then there’s the intersection where both parts of the puzzle are needed” (8, County). A third interviewee noted that staff comfort and safety is an important consideration, and that it can be easier for behavioral health staff to feel safe responding to a mental health emergency when the individual is known to the department:

Philosophically though, we want to align with, if it’s a person who perhaps we know well, somebody who we don’t believe to be a risk in the community but clearly needs help, we believe that probably there wouldn’t need to be a law enforcement presence there. Now, the flip side is also true. If we believe the person may pose a risk, then absolutely, for the safety of our crisis staff, we would absolutely want a police officer there as well. (15, Region)

However, these interviewees often noted that they still need to work out details or formal procedures regarding the role of law enforcement in mobile crisis response.

#### Mobile Crisis-Related Challenges and Concerns

Several interviewees highlighted the challenges that their county faces with respect to mobile crisis teams. Some echoed the previous theme related to **workforce**, emphasizing the intense nature of the work. For example, one interviewee said that they wished they could pay crisis clinicians a “hazard duty pay,” (6, County) as they noticed higher levels of turnover among their crisis clinicians than outpatient clinicians.

Another key challenge related to **geographic constraints**, especially in large or more rural counties. For example, one interviewee noted that mobile crisis response is easier in their population hubs than in far-reaching areas of their county, where limited cell service and winter weather can pose additional challenges. Other interviewees noted that it can take one to two hours to reach some communities in their counties – which means a three- to four-hour round trip.

### Somewhere to Go: Preparedness Related to Crisis Receiving and Crisis Stabilization Services

#### Crisis Receiving and Stabilization Service Structure

Interviewees from about half the jurisdictions reported that they have crisis receiving and/or crisis stabilization services; about 2/3 of these were better-prepared counties. Some of these interviewees noted that their jurisdictions had been working to develop their capacity for crisis stabilization services for quite some time, and they now will be able to leverage those services for 988 callers who require such a level of care. For example, one interviewee noted that they have an access center where individuals can present for treatment or can be brought by a third party (e.g., family, law enforcement), and that this complements other treatment options available in their community (e.g., outpatient or subacute care). When asked if they had sufficient capacity in their region, they said, “Yes, it’s been a struggle, but we’re there. All of our services were gradually built up over time….It’s been a long road” (5, Region). An interviewee from another jurisdiction noted that they first began thinking about stabilization taking place in a standalone treatment center about 10 years ago; over time, the state has been able to establish additional sources of funding to sustain that initial treatment center, add additional centers, and begin establishing programs for certain subpopulations (e.g., crisis respite homes for foster youth).

#### Crisis Stabilization-Related Challenges

However, interviewees from jurisdictions with crisis stabilization options noted that they still experience barriers to providing these services. Some noted that there is still a **reliance on emergency departments**, especially in more rural or remote areas of the jurisdiction. For example, one regional director noted:

With [the crisis stabilization center], it’s available to everyone, but in [one of the counties in this region,] it could be a one-and-a-half hour drive to the center… So when there’s not the crisis centers, the ER are obviously where people go. (4, Region)

There are other challenges that jurisdictions face, including limitations to the **capacity of crisis services and staffing**. In some cases, interviewees noted that they do not have sufficient volume to justify a local crisis stabilization unit or to increase staffing of their existing units, even if it limits the types of patients they can serve. Among regions that do not have crisis stabilization units, many interviewees noted that they rely on an emergency department or resources in neighboring jurisdictions to serve individuals experiencing a mental health emergency. However, even then, it can be challenging to find a unit with available beds.

**Funding** is also an important consideration. As one interviewee reported:

We would love to have it locally, but our local hospital has given it serious consideration, done some benchmarking, and comparing with other hospitals, rural hospitals that have done it, and just feel like the financial risk is great, and they haven’t felt like it’s something they were willing to move forward on. (1, Region)

Relatedly, one interviewee highlighted that access to available crisis stabilization services can be driven in part by insurance:

Many of these services are driven by type of insurance. So, if you have Medicaid or Medicaid managed care, you have more access to these more, I would say, ‘progressive’ services – respite, peer support. For those who have commercial insurance, it’s often a challenge…the rates are too low to accept, or they don’t have access to some of the respite, peer support. (14, County)

Interviewees from jurisdictions with and without crisis stabilization units also highlighted that it is important to think about where crisis stabilization fits within the continuum of care. Interviewees described efforts to provide other types of services, such as respite sites, longer-term residential treatment programs, partial hospitalization or day treatment programs, and outpatient treatment. As one interviewee said:

The 988 rollout’s going to go much more successfully if the community as a whole has better infrastructure to be able to meet needs for people who maybe are in those stabilization units but that don’t necessarily meet inpatient criteria but still need help. They still need support. (15, Region)

### Additional Planning Considerations

#### Funding

The interviews provided an opportunity to learn more about how interviewees’ jurisdictions funded 988 implementation. We asked about the hotline itself and also about mobile crisis response and crisis stabilization services. Several funding models were described, with many jurisdictions drawing on multiple sources of funding to support the continuum of emergency mental health care (i.e., the 988 hotline, mobile emergency response, and emergency stabilization and receiving services). For example, multiple jurisdictions received state funding in some capacity (e.g., fully funding services, partially funding services, funding for one additional staff member) to support 988 implementation and the continuum of care. Specific to the hotline, some jurisdictions received local funding or Vibrant planning grants.

Regarding mobile crisis response, funding sources described included local taxes, grant funding, funding from other local agencies (e.g., Sheriff’s departments, which may have supported co-response models) and state agencies, and funding from the American Rescue Plan Act. Finally, in terms of mental health emergency stabilization services, interviewees from several jurisdictions reported that Medicaid and private insurance were used to fund services. Other sources included funds from a local hospital or tax-levied funds, and funding allocated at a state level for behavioral health services.

#### Use of Peer Support Specialists in the Mental Health Emergency Healthcare Continuum

Peer support specialists are essential community staff members who have lived experience and provide non-clinical support as an integrated team member in mental health crisis situations. During the qualitative interviews, we asked interviewees about the use of peers in crisis response. About a third of interviewees reported that peer support specialists have a specific role in their crisis response model. Interestingly, the way in which peer supports are used is inconsistent and varies even within jurisdictions; for example, one interviewee reported,

Some of the Lifeline centers, I want to say all of them, likely have peers who are involved with them. Some I think more than others, depending on where they are, because each are run differently. (7, County)

Interviewees from most agencies that do not currently use peer support staff in their crisis continuum acknowledged the benefit of employing peers, and many described plans to include a peer support specialist model in their continuum of emergency mental health care services. At the same time, some interviewees expressed concern about the feasibility of developing a peer support worker model, noting the nationwide behavioral health workforce issues, a loss of funding due to the low billable rate, and a lack of guidance around hiring peer support (e.g., through a subcontract or crisis agency). One interviewee said,

It’s difficult in the moment because we have such tremendous staffing issues, just in general. It’s difficult to think about – and it’s probably true that it’s easier to hire the peer workforce than it is licensed clinicians, for instance…But it’s also, I know from experience, that it’s challenging to expect people to work a regular shift and then be responding to crisis in addition. So building a team that has that dual role – so yeah, I think it would be amazing to have a peer or peers as part of the crisis response team. (13, County)

In addition, though not all interviewees described using peer support specialists in their crisis continuum, many noted that peer support staff are integrated into other local behavioral health services. For example, some jurisdictions have peer-run organizations that are involved in outreach, community initiatives, and crisis assessment. Other interviewees described having peers involved in post-crisis response follow-up, training of behavioral health staff, or operation of a warm line.

#### Addressing the Needs of Special Populations

All of the interviewees noted that their agency is aware of the specialized needs of groups such as military veterans, LGBTQ + individuals, or youth. Some interviewees indicated that they have developed special services for youth, such as mobile emergency response teams specifically focused on youth. A small number said that they will be able to leverage local service providers with expertise in serving subpopulations (e.g., LGBTQ + populations, veterans). However, many noted that there are not specific plans in place to serve these groups.

We also asked interviewees whether there were additional communities or populations that they thought might be more challenging to serve via a call center, mobile crisis response team, or crisis stabilization services. Additional populations included those who may be hesitant to call 988 and engage with government-funded services or services outside of their community, such as Amish communities, immigrants and migrant workers, and those with mixed documentation status. Other communities were described as more challenging to serve for logistical reasons; for example, interviewees from large rural areas noted that mobile crisis teams can have a difficult time reaching certain areas of their counties in a timely manner. Another described challenges finding translators to serve those who do not speak English. Although there was a general lack of concrete plans to improve service provision to underserved communities, nearly all interviewees recognized the need to do so.

### Strengths, Limitations, and Near Term Priorities

#### Primary Limitations and Challenges to 988 Implementation Plans

Our question regarding primary limitations was intended to identify the areas that interviewees have found to be most challenging as they prepare for the launch of 988. The most common response was **workforce and staffing challenges**. Interviewees highlighted workforce shortages and difficulties both hiring and retaining staff. This challenge seemed to be common across different types of jurisdictions; for example, an interviewee in a rural area noted that it can be difficult to find staff willing to relocate to their area compared to larger metropolitan areas. However, an interviewee from a state-level agency also indicated that having sufficient workforce is a barrier throughout their state. As another county interviewee stated:

Community negotiations and partnerships, they’re actually easy compared to finding and hiring and keeping staff. (6, County)

The next most common challenge identified by interviewees was **funding or lack of resources**. This issue was raised most often by interviewees from less-prepared jurisdictions, though one better-prepared jurisdiction also highlighted a lack of financial support for their hotline and mobile crisis services. Funding was often raised in conjunction with staffing issues. For example, as one interviewee stated:

I think, if we were adequately funded, I believe…we would have the skillset to do an excellent job. (15, Region)

A small number of interviewees highlighted other limitations of their 988 plans, including a **lack of unified planning and strong sources of guidance** and a **need to ensure proper handoff and care coordinatio**n. For example, one interviewee expressed concern about what might happen if a Lifeline call was routed out of state, and whether there would be sufficient knowledge of local resources to make an appropriate referral. A small number of interviewees also highlighted challenges **reaching certain areas of their jurisdiction** as one of their primary limitations; this was particularly an issue in more rural jurisdictions.

#### Primary Strengths of 988 Implementation Planning

We also asked interviewees to describe the primary strength of their jurisdictions’ 988 plans. This question was not intended to capture all aspects of a “successful” planning process, but rather to understand what interviewees perceived as one or two key strengths of their jurisdictions’ plans. The most common response was **collaboration and partnerships**. Both better-prepared and less-prepared counties noted that their interagency collaborations have been a critical aspect of their planning, including partnerships with behavioral health providers, other county agencies (e.g., child welfare, law enforcement, criminal justice agencies), hospitals, and local leadership.

Some interviewees also described the **capabilities and expertise of their current behavioral health providers** as a key strength. For example, one interviewee noted that having a local provider as the 988 hotline provider will ensure that they are familiar with local resources, which will benefit callers. Similarly, another interviewee described their 988 and mobile crisis provider as “well-experienced,” noting:

…[it] really has the structure, stability, resources to be able to provide the service… That’s one of the reasons we selected this provider. They have their fingers in pretty much all we do. (14, County)

Another praised the skill of their long-standing mental health emergency staff:

We have people who have worked in crisis intervention for decades who clearly know the law around working with people under the [State Mental Health Legislation]. They also are very gifted in knowing what resources exist in the community and how to access those resources. They’re good at doing follow-up after a call if the person has been released. So I feel like we have a good pool of people who know their jobs and do them well. (15, Region)

A small number of interviewees also indicated that they have da**ta to support planning and decision-making**, which they perceived as a strength.

#### Priorities Before the Launch of 988

Finally, we asked interviewees to reflect on the most important goal for their jurisdiction prior to July 16, when 988 was to launch. Nearly half of the interviewees noted that there needed to be **more education of the public** to ensure they are aware of 988. Some interviewees described local efforts they planned to initiate from within their agency (e.g., promoting 988 on their agency’s website and through social media), but one interviewee also noted that they wished there were more formal federal guidance to states on how to raise awareness of 988 at the local level. A small number of interviewees indicated that their jurisdictions were focused on **formalizing policies and procedures** related to 988 and the continuum of care—for example, establishing clear protocols, established lines of communication, and memoranda of understanding (MOUs) between agencies. This also included developing plans for the interface between 988 and 911 or local crisis lines. A small number of interviewees also expressed a desire for their agency to have greater **involvement in state or regional planning efforts**:

So far, I’m not at that table so I’m not sure what’s happened. I don’t even know who should be at that table, and whose responsibility it is to convene the table. 1 (3, County)

Other responses included the need to **formalize additional funding sources and ensure there was adequate workforce**. A summary of interviewee perceptions of challenges, strengths, and near-term priorities appears in Table 2.

**Table 2:** Summary of perceived challenges, strengths, and near-term priorities for 988 planning

Theme	Summary of responses
Challenges to 988 implementation planning	• Workforce shortages • Difficulty retaining staff • Lack of funding • Lack of coordinated planning efforts • Need for concrete guidance • Concerns about care coordination
Strengths of 988 implementation planning	• Collaborations and partnerships with local agencies and providers • Capable behavioral health providers with needed expertise • Data to support decision-making
Priorities before the launch of 988	• Raising public awareness of 988 • Formalizing policies and procedures related to 988 and the continuum of emergency mental health care • Participating in regional or state planning efforts • Formalizing funding for 988 • Improving staffing levels for 988 and the continuum of care

## Discussion

The implementation of 988 has been heralded as a critical milestone for transforming emergency mental health care in the United States (SAMHSA, 2022). At its most basic level, the launch of this easy-to-remember telephone number means that more people in need of emergency mental health services may be able to access needed care. However, for many states and counties, it has also presented an opportunity to prepare for an increase in call volume and bolster their continuum of emergency mental health services. This study aimed to describe how jurisdictions have been preparing for the launch of 988 and the implications for their mobile crisis and crisis stabilization services. In addition, by focusing on jurisdictions that described themselves as more- and less-prepared for 988 implementation, our goal was to identify promising practices and common challenges faced by these jurisdictions.

The most notable distinction that we found between more- and less-prepared jurisdictions was that better-prepared jurisdictions already appeared to have a more robust continuum of emergency mental health services. For example, these jurisdictions already had a local Lifeline center, 24/7 mobile crisis teams that could respond within 30 min, and local crisis stabilization services. Though there may have been efforts to bolster these existing services – for example, by obtaining grants to increase the capacity of their Lifeline center – these jurisdictions could capitalize on existing resources and services.

At the same time, we found that there were some perceived challenges that affected both types of jurisdictions. One such barrier pertained to workforce and capacity, the challenges hiring and retaining staff with necessary credentials, particularly for mobile crisis and stabilization services. In part, this issue is indicative of the larger mental health workforce shortage (Covino, 2019), a key issue limiting access to behavioral health services. But as some interviewees pointed out, burnout is another concern that can affect retention among existing emergency mental health staff. It is likely that a multipronged strategy will be needed to address this concern and ensure adequate staffing for emergency mental health services. First, to mitigate burnout, it is important that mental health staff have sufficient support from colleagues and supervisors, appropriate training and resources, and manageable workload (SAMHSA, 2021a; Warlick et al., 2021; Zivin et al., 2022). Second, jurisdictions could more intentionally integrate peer support staff into their continuum of crisis care. The SAMHSA National Guidelines for Behavioral Health Crisis Care note that peers play important roles throughout the continuum of care, recommending that nearly 50% of the mobile crisis workforce could be staffed by peer support specialists (SAMHSA, 2020).

Another perceived challenge that affected both more- and less-prepared jurisdictions pertains to geographical considerations. Regarding crisis hotlines, concerns were raised about having calls answered by a call center outside of the local area, and especially outside the state. Though the Lifeline aims for 90% of calls to be answered by an in-state call center, a recent analysis based on 2020 data, before 988, found that only seven states achieved this benchmark, and the in-state answer rate was as low as 10% in one state (Purtle et al., 2022). At the same time, there is evidence that many calls do not require a local referral (e.g., Ramchand et al., 2017); therefore, it will be important to monitor these trends now that 988 has been implemented to determine what number of callers need a local referral and how to best meet their needs. In addition, rural areas struggled with providing timely mobile crisis and crisis stabilization services to more remote areas. Though some resources, including the SAMHSA guidelines, provide some guidance for rural and frontier communities, more-tailored guidance for those types of jurisdictions could be valuable. It will also be important to explore challenges faced by areas of the country known to have more limited availability of crisis services, including Southeast, Southwest, and Western states (Kalb et al., 2022).

Funding was another key challenge, especially among less-prepared jurisdictions. Leading up to the rollout of 988, the federal government allocated approximately $282 million for strengthening network operations and strengthening local crisis call center capacity, and numerous grants were been awarded through Vibrant Emotional Health, the nonprofit administrator of 988 (SAMHSA, 2021c). However, these sources of funding are not sustainable in the long run. Looking to 911 as a corollary, most states have embedded a surcharge for 911 callers, the proceeds of which are directed to support 911 and emergency service operations (Hartsig, 2017). If more states imposed comparable surcharges for 988, it could be an important funding source (Fix et al., 2023). Jurisdictions could also look to states that have developed effective models drawing on Medicaid funds, such as Arizona (Hogan & Goldman, 2021).

As jurisdictions build their continuum of emergency mental health care, they are also working to delineate the roles of mental health staff versus law enforcement in responding to behavioral health emergencies. Clinician-led models, such as the CAHOOTS model, have received national attention as an option for reducing the role of law enforcement in responding to people experiencing a mental health crisis (Eugene Police Crime Analysis Unit, 2022). However, many interviewees noted that there might be a more formal role for law enforcement in their jurisdiction. There are promising approaches that might ensure that a situation doesn’t escalate when law enforcement is involved in a behavioral health matter, such as crisis intervention training or co-responder models (Pinals & Edwards, 2021). However, there is little rigorous evidence of the effectiveness of these approaches (Puntis et al., 2018; Rogers et al., 2019).

There is also room for growth in the extent to which jurisdictions can address the needs of special populations (e.g., veterans, immigrants) through their continuum of emergency behavioral health care. Importantly, many of these groups may be especially at-risk for mental health emergencies (Johns et al., 2020; Kang et al., 2015), highlighting the importance of ensuring that providers are well trained to serve these groups and that culturally competent providers are available in the local community. Jurisdictions must also seek to create inroads that increase individuals’ willingness to use mental health emergency services in the first place. Collaborating with credible messengers and members of these difficult-to-reach communities may be one way to address this concern.

Our interviewees also provided insight into perceived strengths of jurisdictions’ 988 planning process, including developing collaborations and partnerships with local agencies and providers, having well-qualified providers, and collecting relevant data to guide decisions regarding staffing and needed programming. This includes data regarding the volume of individuals using services at each stage in the continuum; information regarding wait times (e.g., number of minutes for calls to be answered, number of minutes for a mobile crisis unit to reach an individual, number of days for an individual to schedule an appointment); and a census of available behavioral health treatment slots, including crisis stabilization and other services (National Action Alliance for Suicide Prevention Crisis Services Task Force, 2016). Because these data may be housed within different agencies, establishing the infrastructure to support data integration and data sharing (e.g., memoranda of understanding, data sharing agreements) is key. At the same time, it is important to acknowledge that it is only with time that we will be able to determine if these truly were the keys to successful 988 planning, as our interviews drew on individual perceptions before the launch of 988.

This study has certain limitations. First, we had a relatively small sample size and we intentionally selected interviewees from more- and less-prepared jurisdictions. Therefore, these findings are not necessarily generalizable to other jurisdictions, in particular those that fall somewhere between those two extremes. However, generalizability is not the goal of a qualitative study such as this, and the richness of our data illuminates nuances about 988 preparation and the continuum of emergency mental health care that would be difficult to obtain with quantitative data alone. Second, as described, we identified potential interviewees based on their responses to an earlier survey. Only 13% of survey respondents expressed willingness to participate in an interview, and those interviewees represented only ten states. We included multiple representatives from some states to ensure that our sample was large enough to reach saturation. That said, when we included two interviewees from a single state, we aimed to select people who represented jurisdictions that were diverse in some way – for example, in one state, we included an interviewee from a county with a population of more than 900,000 and an interviewee from a county with a population less than 50,000. Finally, this study was conducted in the months leading up to the launch of 988, and therefore responses do not reflect learnings that may have occurred in the early days of 988 implementation.

However, this study also makes an important contribution to our understanding of how 988 may shape the country’s approach to providing emergency mental health services. In particular, this study provides in-depth insights into the common challenges faced across jurisdictions as they work to strengthen their continuum of emergency mental health services, as well as the characteristics that seem to distinguish more-prepared jurisdictions from less-prepared jurisdictions. It will be important for future work to explore how the launch of 988 affects the provision of emergency mental health services across the country, and to continue to identify best practices and innovative strategies for addressing the needs of individuals experiencing a crisis.

## Footnotes

1 Note that this quote was paraphrased due to poor recording quality during this interview.
